# Predictors and outcomes of acute pulmonary embolism in COVID-19; insights from US National COVID cohort collaborative

**DOI:** 10.1186/s12931-023-02369-7

**Published:** 2023-02-21

**Authors:** Muhammad H. Gul, Zin Mar Htun, Vinicio de Jesus Perez, Muhammad Suleman, Samiullah Arshad, Muhammad Imran, Mahender Vyasabattu, Jeremy P. Wood, Michael Anstead, Peter E. Morris

**Affiliations:** 1 Internal Medicine Department, University of Kentucky, MN 602, H Building, 1000 S Limestone, Lexington, KY 40506 USA; 2 Pulmonary Critical Care Department, University of Maryland, Baltimore & National Institute of Health Sciences, Baltimore, MD USA; 3 Pulmonary Critical Care Department, Stanford University, Stanford, CA USA; 4 Cardiology Department, Peshawar Institute of Cardiology, Peshawar, Pakistan; 5 Cardiothoracic Surgery Department, Armed Institute of Cardiology Rawalpindi, Rawalpindi, Punjab Pakistan; 6 Division of Cardiovascular Medicine, The Gill Heart and Vascular Institute, University of Kentucky, Lexington, KY USA; 7 Saha Cardiovascular Research Center, University of Kentucky, Lexington, KY USA; 8 Department of Molecular and Cellular Biochemistry, University of Kentucky, Lexington, KY USA; 9 Pulmonary Critical Care Department, University of Kentucky, Lexington, KY USA; 10 Pulmonary Critical Care Department, University of Kentucky, Lexington, KY USA

**Keywords:** Acute pulmonary embolism, COVID-19, SARS-CoV-2, d-Dimer, Mortality

## Abstract

**Objectives:**

To investigate whether COVID-19 patients with pulmonary embolism had higher mortality and assess the utility of d-dimer in predicting acute pulmonary embolism.

**Patients and methods:**

Using the National Collaborative COVID-19 retrospective cohort, a cohort of hospitalized COVID-19 patients was studied to compare 90-day mortality and intubation outcomes in patients with and without pulmonary embolism in a multivariable cox regression analysis. The secondary measured outcomes in 1:4 propensity score-matched analysis included length of stay, chest pain incidence, heart rate, history of pulmonary embolism or DVT, and admission laboratory parameters.

**Results:**

Among 31,500 hospitalized COVID-19 patients, 1117 (3.5%) patients were diagnosed with acute pulmonary embolism. Patients with acute pulmonary embolism were noted to have higher mortality (23.6% vs.12.8%; adjusted Hazard Ratio (aHR) = 1.36, 95% CI [1.20–1.55]), and intubation rates (17.6% vs. 9.3%, aHR = 1.38[1.18–1.61]). Pulmonary embolism patients had higher admission D-dimer FEU (Odds Ratio(OR) = 1.13; 95%CI [1.1–1.15]). As the d-dimer value increased, the specificity, positive predictive value, and accuracy of the test increased; however, sensitivity decreased (AUC 0.70). At cut-off d-dimer FEU 1.8 mcg/ml, the test had clinical utility (accuracy 70%) in predicting pulmonary embolism. Patients with acute pulmonary embolism had a higher incidence of chest pain and history of pulmonary embolism or deep vein thrombosis.

**Conclusions:**

Acute pulmonary embolism is associated with worse mortality and morbidity outcomes in COVID-19. We present d-dimer as a predictive risk tool in the form of a clinical calculator for the diagnosis of acute pulmonary embolism in COVID-19.

**Supplementary Information:**

The online version contains supplementary material available at 10.1186/s12931-023-02369-7.

## Introduction

As of Feb 2023, over 6 million deaths have been attributed to COVID-19 worldwide [1]. COVID-19 associated coagulopathy confers an increased risk of microvascular and macrovascular thrombosis [2]. Histological examination of pulmonary vessels in COVID-19 is distinguished from other viruses by widespread micro-thrombosis [3]. In the meta-analysis, high incidence rates of pulmonary embolism, 16.5% (95% CI: 11.6–22.9), have been noted in COVID-19 [4]. Relatively small studies have reported worse outcomes associated with PE in COVID-19; however, to our knowledge, no clear association of increased mortality associated with PE in COVID-19 has yet been reported [5, 6]. We wished to assess whether COVID-19 patients with diagnosed PE have increased mortality when compared to COVID-19 patients without PE.

The screening of acute PE in COVID-19 patients remains an important clinical question to date. Our traditional clinical predictors of screening for acute PE such as tachycardia, dyspnea, or hypoxia are confounded by the clinical presentation of COVID-19 pneumonia. The conventional scoring systems, such as Well’s criteria, have been noted to be non-discriminatory (AUC 0.54) in predicting the risk of acute PE in COVID-19 patients [7, 8]. Previous studies have pointed towards the possible role of d-dimer in the assessment of COVID-19 patients for screening of PE; however, no clear prediction scheme has been devised [5, 9, 10]. d-dimer is generated as a result of the lysis of blood clots as a part of the normal healing process. d-dimer is released when the cross-linked fibrin undergoes lysis by the plasmin-mediated degradation process [11]. d-dimer would be released in the blood during the breakdown process of a blood clot, as would be the case with pulmonary embolism. Using a national database, the National Collaborative COVID-19 cohort (N3C), we intended to develop an easy-to-use clinical calculator using d-dimer for predicting the risk of acute PE in hospitalized COVID-19 patients [12]. We also studied the commonly used clinical laboratory parameters to formulate the pathogenesis of acute PE in COVID-19.

## Methods

### Data source

The National Collaborative COVID-19 cohort (N3C) is the largest US database registry for COVID-19 patients and their representative controls [13, 14]. The N3C Data Enclave is approved under the authority of the NIH Institutional Review Board. An Institutional data-use agreement between the University of Kentucky and N3C data Enclave provided access to the de-identified database. N3C harmonizes its dataset based on four heterogeneous sources (ACT Network, TriNetX, OHDSI, PCORNet) and provides a unified platform within the N3C enclave for statistical analysis. The N3C cohort represents a rich, highly granular, ethnically, and geographically diverse dataset from clinical sites throughout the US (including Southeast, Midatlantic, and Midwest USA). The N3C cohort includes patients with any encounter after Jan 1, 2020, based on one of a set of SARS-CoV-2 laboratory tests or diagnostic codes. Further details about the N3C cohort are listed in the data source Additional file 1.

### Measures, definition, and outcomes

**Fig. 1:**
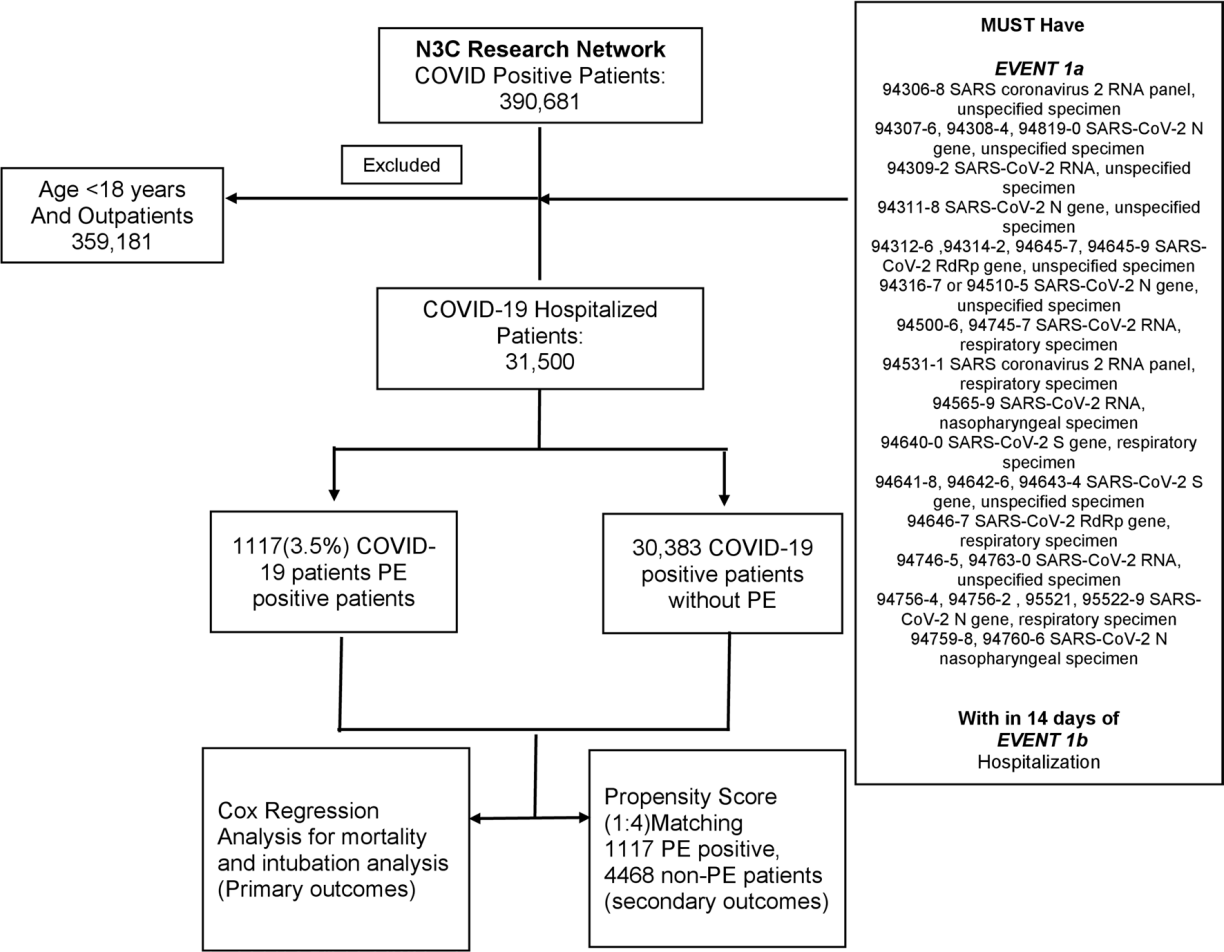
Flow chart describing the schema of the study population. Primary outcomes included mortality and intubation analysis. Secondary outcomes included length of stay, chest pain incidence, heart rate, history of deep vein thrombosis or PE, and comparison of admission laboratory parameters (ferritin, C-reactive protein [CRP], d-dimer fibrinogen equivalent units [FEU], and d-dimer units [DDU], fibrinogen, lactate dehydrogenase [LDH], and lymphocyte count). Separate propensity score matched analysis was performed for each lab parameter

The N3C database was accessed on Jan 7, 2021 (the time period of encounters from Jan 1, 2020, to Jan 7, 2021), which provided data from 29 clinical sites. We performed a retrospective observational cohort analysis using N3C, including individuals aged ≥ 18 years, with a positive SARS-CoV-2 PCR test within 2 weeks of hospitalization (Fig. 1). Based on the diagnosis of acute PE during the admission encounter, hospitalized COVID-19 patients were divided into PE and non-PE groups. Hospitalized COVID-19 patients are not universally screened with CT pulmonary embolism in the clinical setting. Hence, the small possibility of undiagnosed pulmonary embolism exists in this unscreened population—designated as non-PE in our study, based on the absence of acute pulmonary embolism diagnosis in the database. Baseline characteristics included demographics (age, sex, and race), comorbidities (atherosclerosis, congestive heart failure, diabetes, hypertension, atrial fibrillation, chronic kidney injury, acute kidney injury, chronic obstructive pulmonary disease, obstructive sleep apnea, hypothyroidism, obesity, anemia, nicotine dependence, malignancy, and sepsis) and inpatient medications use (steroids, remdesivir, tocilizumab, hydroxychloroquine). The continuous variables, if normally distributed, were reported as means ± SD or as medians and interquartile ranges, if not normally distributed. The primary outcomes were 90-day mortality rate and 90-day intubation rate from the time of hospitalization. Secondary analyzed parameters included length of stay, chest pain incidence, heart rate, history of deep vein thrombosis or PE, and comparison of admission laboratory parameters (ferritin, C-reactive protein [CRP], d-dimer fibrinogen equivalent units [FEU], and d-dimer units [DDU], fibrinogen, lactate dehydrogenase [LDH], and lymphocyte count). The secondary analyzed parameters included a history of deep vein thrombosis or PE, as we wanted to evaluate whether it is an associated risk factor for PE in COVID-19. The data accessed on Jan 7, 2021, was used for both primary and secondary outcomes analysis. In the clinical setting, two different d-dimer assays (d-dimer FEU and d-dimer DDU) are widely utilized, depending on the lab preference of the clinical site. The Fibrinogen Equivalent Unit (FEU) reporting of d-dimer levels is based on the molecular weight of fibrinogen (340 kDa) and the d-Dimer Unit (DDU) reporting of d-dimer levels is based on the molecular weight of d-dimer (195 kDa), which is about half that of fibrinogen. We performed a separate analysis for each assay.

### Statistical analysis

For the primary outcomes study, a multivariable time-dependent cox proportional hazard regression model was adjusted for demographics, comorbidities, and medication use as described earlier, to compare the mortality and intubation outcomes in the acute PE and non-PE hospitalized COVID-19 patient groups [15]. Patients were followed up until the time of an event or censored at 90 days for mortality or intubation outcomes. For the intubation outcomes analysis, in the PE group, patients who had PE diagnosed after the day of intubation were excluded. Overall survival and hazard for intubation were evaluated with the log-rank test. The Kaplan Meier and hazard curves were constructed for mortality and intubation outcomes, respectively. The covariates in the cox-proportional hazard model were carefully chosen based on existing evidence of their association with COVID-19 or all-cause mortality [16]. Further details about the assumption of proportionality of hazards in cox regression are listed in the Additional file 1 biostatistical methods. For the secondary outcomes study—which required comparison of absolute values post-matching—a separate adjusted 1:4 propensity score matching with the logistic regression model, greedy nearest neighbor, without replacement and caliper of 0.2 (Additional file 1: methods) was used to construct PE and non-PE groups (Fig. 1). The propensity score matching was preferentially used for the analysis of secondary parameters so that post-matching, the secondary parameters can be visually compared which can signify clinical significance (Fig. 4) and ROC curves can be constructed for d-dimer on the matched data (Additional file 1: Figs. S4, S5). 1:4 propensity score matching was performed for a larger number of non-PE patients than PE patients in order to reduce data wasting and hence, improve study power [17–19]. The covariates in the propensity score-matched model were either related to the outcome due to their association with COVID-19 or treatment (PE in COVID-19) [19, 20]. The covariates in the propensity score-matched analysis were also demographics (age, sex, and race), comorbidities (atherosclerosis, congestive heart failure, diabetes, hypertension, atrial fibrillation, chronic kidney injury, acute kidney injury, chronic obstructive pulmonary disease, obstructive sleep apnea, hypothyroidism, obesity, anemia, nicotine dependence, malignancy, and sepsis) and inpatient medications use (steroids, remdesivir, tocilizumab, hydroxychloroquine), similar to the covariates used in the primary outcomes study in cox-regression analysis. The standardized mean deviations were calculated to compare the balance between the two matched groups (Additional file 1: Fig. S1). They were then analyzed to compare the length of stay, chest pain incidence, heart rate (first reading recorded), and deep vein thrombosis or PE history. The propensity score matched results were analyzed via univariate logistic regression to calculate odds ratio. Since the admission labs were not present in all the patients, a separate adjusted propensity score-matched analysis was performed for each lab parameter (Additional file 1: Table S1). ROC curves were constructed to determine the characteristics of admission d-dimer FEU and d-dimer DDU in predicting acute PE in hospitalized COVID-19 patients. The sensitivity, specificity, positive predictive value, and accuracy of d-Dimer FEU and d-Dimer DDU to predict pulmonary embolism at different threshold levels are recorded in Additional file 1: Tables S2 and S3, which were subsequently exported to construct an easy-to-use calculator which was published online by our group [21]. The COVID-19 d-dimer calculator thus provides sensitivity, specificity, positive predictive value and accuracy of d-dimer FEU and d-dimer DDU to predict PE for various threshold levels [21]. The ROC optimal cut off value was selected based on the accuracy (accuracy ≥ 70%), as has been suggested previously by Korevaar et al. [22] Missing data (age 1.25%, race 12.9%, gender 0.5%) was imputed with the multivariate imputation by chained equations (MICE) throughout the data analysis. Significance was defined as p < 0.05 when using a two-tailed test. The odds ratio for categorical variables was calculated using Altman’s method on MedCalc software 20.013 [23, 24]. Odds ratio for continuous variables was calculated via univariate logistic regression. Further details about the statistical methods are provided in the Additional file 1. The analysis was performed within the Palantir Foundry, hosted within the cloud-based N3C enclave using Python 3.6 (Python Software Foundation) and R 3.5.1(R project for statistical computing).

## Results

As of Jan 7, 2021, 390,681 patients were identified as COVID-19 positive based on PCR testing. Out of these, 31,500 COVID-19 adult patients were hospitalized. 1,117(3.5%) of these patients were diagnosed with acute PE during their admission. Primary propensity score matching yielded 1,117 PE patients and 4,468 non-PE patients (Fig. 1). 128 patients had PE diagnosed after the day of intubation and were excluded from the intubation outcomes analysis. Acute PE was diagnosed in 302 (9.7%) of 3,116 intubated patients (including those who were diagnosed post-intubation).

### Baseline characteristics

Acute PE diagnosis in COVID-19 patients was recorded at median 0, interquartile range (IQR) 0–3 days post-admission. In our univariable analysis of COVID-19 patients, older patients (age 62 vs. 57 years; odds ratio (OR) 1.014, 95% CI [1.011–1.018]), and males (58% vs. 51%; OR 1.35 [1.20–1.52]) were more likely to have acute PE. A higher incidence of acute PE was noted in African Americans (OR 1.34 [1.18–1.52]) and Asian Americans (OR 1.45 [1.04–2.2]). Patients with acute PE had a higher prevalence of comorbidities, notably atrial fibrillation, hypertension, diabetes, congestive heart failure, obstructive sleep apnea, obesity, anemia, chronic kidney disease, malignancy. Higher incidence of DVTs (9% vs. 0.2%; OR 46.16 [33.65–63.3]), cerebrovascular diseases (5.8% vs. 3.5%; OR 1.66 [1.28–2.18]), acute encephalopathy (11.2% vs. 6%; OR 1.98 [1.63–2.39]), acute kidney injury (42.2% vs. 27.8%; OR 1.89 [1.67–2.14]) and sepsis (34.5 vs. 22.1%; OR 1.85 [1.63–2.1]) was also noted in acute PE patients. Patients with acute PE were more likely to receive treatments such as steroids, remdesivir, and tocilizumab (Table 1).

**Table 1:** Baseline characteristics of patients with and without acute pulmonary embolism

	Pulmonary embolism COVID-19 patients (n = 1117)	Non-pulmonary embolism COVID-19 patients (n = 30,383)	Odds ratio, p value
Age ± SD	62 ± (15.1)	57 ± (19.7)	1.014 (1.011–1.018), p < 0.001
Females	474 (42%)	15,180 (49%)	0.73 (0.65–0.83), p < 0.001
Race			
Caucasians	555 (49%)	15,513 (51%)	0.94 (0.84–1.06), p = 0.36
African American	378 (33%)	8361 (27.5%)	1.34 (1.18–1.52), p < 0.001
Asian American	38 (3.4%)	719 (2.3%)	1.45 (1.04–2.2), p = 0.02
Others	168 (15%)	5790 (19%)	0.75 (0.63–0.88), p < 0.001
Atrial fibrillation/flutter	168 (15%)	3685 (12.1%)	1.28 (1.08–1.51), p = 0.003
HTN	677 (60.6%)	15,817 (52%)	1.41 (1.25–1.60), p < 0.001
Diabetes	393 (35.1%)	8317 (27.3%)	1.44 (1.27–1.63), p < 0.001
Atherosclerosis	171 (15.3%)	4350 (14.3%)	1.08 (0.91–1.27), p = 0.35
Acute kidney injury	472 (42.2%)	8459 (27.8%)	1.89 (1.67–2.14), p < 0.001
Chronic kidney disease	156 (13.9%)	3588 (11.8%)	1.21 (1.02–1.44), p = 0.02
Congestive heart failure	234 (20.9%)	5085 (16.7%)	1.31 (1.13–1.52), p < 0.001
Chronic obstructive pulmonary disease	116 (10.3%)	3098 (10.19%)	1.02 (0.83–1.24), p = 0.83
Obstructive sleep apnea	178 (15.9%)	3922 (12.9%)	1.27 (1.08–1.50), p = 0.003
Sepsis	386 (34.5%)	6742 (22.1%)	1.85 (1.63–2.1), p < 0.001
Hypothyroidism	148 (13.2%)	3601 (11.8%)	1.13 (0.95–141), p = 0.15
Obesity	339 (30.3%)	7251 (23.8%)	1.39 (1.22–1.58), p < 0.001
Malignancy	68 (6%)	766 (2.5%)	2.5 (1.94–3.23), p < 0.001
Anemia	366 (32.7%)	7106 (23.3%)	1.59 (1.40–1.81), p < 0.001
Nicotine dependence	107 (9.5%)	3118 (10.2%)	0.92 (0.75–1.13), p = 0.45
Acute DVT of extremities	102 (9%)	66 (0.2%)	46.16 (33.65–63.3), p < 0.001
Acute encephalopathy	125 (11.2%)	1819 (6%)	1.98 (1.63–2.39), p < 0.001
Stroke/cerebrovascular diseases	65 (5.8%)	1077 (3.5%)	1.66 (1.28–2.18), p < 0.001
Steroids	610 (54%)	11,653 (38.3%)	1.93 (1.71–2.18), p < 0.001
Remdesivir	311 (27%)	5746 (18.9%)	1.65 (1.45–1.89), p < 0.001
Tocilizumab	32 (2%)	410 (1.3%)	2.15 (1.49–3.10), p < 0.001
Hydroxychloroquine	86 (7.6%)	2020 (6.6%)	1.17 (0.93–1.46), p = 0.17

### Primary outcomes

**Fig. 2:**
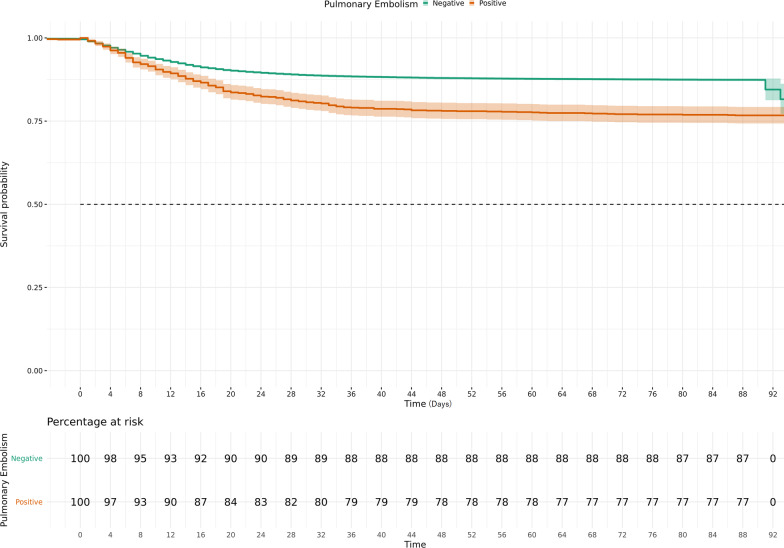
Kaplan Meier estimates of 90-day Survival in the COVID-19 patients with and without acute pulmonary embolism. Acute pulmonary embolism is associated with increased mortality in COVID-19 patients

**Fig. 3:**
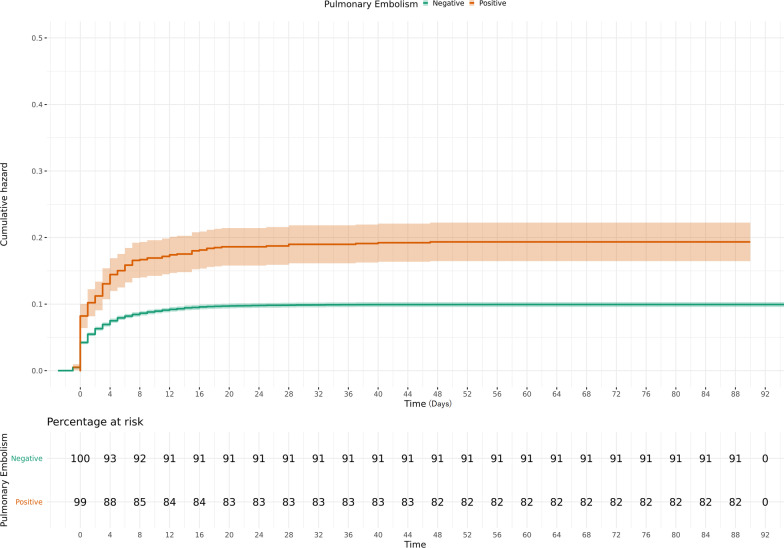
Hazard curve of mechanical ventilation in the COVID-19 patients with and without Acute Pulmonary Embolism. Acute pulmonary embolism is associated with increased mechanical ventilation rates in COVID-19 patients

COVID-19 patients with acute PE had higher 90-day mortality (264 (23.6%) vs. 3918(12.8%), adjusted hazard ratio (aHR) = 1.36 [1.20–1.55] and 90-day intubation rates (174 (17.6%) vs. 2874 (9.3%), aHR = 1.38 [1.18–1.61] when compared to non-PE COVID-19 patients (p < 0.001 for both by log-rank test) (Figs. 2, 3). The unadjusted hazard ratio (HR) for mortality outcome was 1.92 [1.7–2.18] and 1.94 [1.66–2.26] for intubation outcome (p < 0.001 for both). Time from hospitalization to intubation was recorded at a median of 1 [IQR 0–4] days for both groups (OR 0.99[0.98–1.01]). For PE patients, who were intubated, the time difference between PE diagnosis and intubation since hospitalization was noted at 0 [IQR − 12 to 1.75] days (Additional file 1: Fig. S2). Hospital length of stay for patients who died was 14 [IQR 6.5–26] days for PE patients vs. 11 [IQR 5–20] days for non-PE patients (OR 0.99 [0.99–1]).

### Secondary parameters

Hospital length of stay was longer for acute PE patients (median 9 [IQR 5–18] vs. 6 [3–11] days; OR 1.02 [1.02–1.03]. A history of DVT or PE (8.2% vs. 1.7%; OR 5.11 [3.75–6.98]) and incidence of chest pain (8.3% vs. 5.5%; OR 1.55 [1.21–1.99]) were more common in patients with acute PE (Table 2).

**Table 2:** Outcomes of COVID-19 patients with and without acute pulmonary embolism

	Pulmonary embolism positive^a^	Pulmonary embolism negative^b^	aHR/OR^c^ p value
Mortality	264 (23.6%)	3918 (12.8%)	1.36 (1.20–1.55), p < 0.001
Intubation	174 (17.6%)	2874 (9.3%)	1.38 (1.18–1.61), p < 0.001
History of pulmonary embolism/DVT	92 (8.2%)	77 (1.7%)	5.11 (3.75–6.98), p < 0.001
Chest pain	93 (8.3%)	246 (5.5%)	1.55 (1.21–1.99), p < 0.001
Length of stay (days)	9 (5–18)	6 (3–11)	1.02 (1.02–1.03), p < 0.001
CRP (mg/dl)	89.95 (41.98–176.93)	76.8 (31–152)	1.001 (1.0009–1.002), p < 0.001
Ferritin (ng/ml)	665 (292–1292)	556 (256–1181)	1 (0.99–1), p = 0.57
d-dimer FEU (mcg/ml)	1.98 (0.95–6.38)	0.97 (0.54–1.87)	1.13 (1.1–1.15), p < 0.001
d-dimer DDU (mcg/ml)	1.03 (0.44–3.74)	0.42 (0.25–0.80)	1.13 (1.06–1.21), p < 0.001
Fibrinogen (mg/dl)	539 (393–684)	553 (430–681)	0.999 (0.998–0.999), p = 0.03
LDH (units/l)	406 (293–567)	341 (253–478)	1.0003 (1.00001–1.0006), p = 0.001
Lymphocytes (1000/ml)	1.04 (0.67–1.59)	0.96 (0.65–1.4)	1.04 (0.99–1.09) p = 0.101
Heart rate (beats/min)	91 (77–104)	86 (76–100)	1.009 (1.004–1.013), p < 0.001

Data are n (%) or median (IQR 25–75th percentile)

^a^Total no of pulmonary embolism positive COVID-19 patients for mortality and secondary outcomes (history of pulmonary embolism/DVT, chest pain, length of stay, CRP, ferritin, D-dimer FEU and DDU, fibrinogen, LDH, lymphocytes, Heart rate) are 1117. For intubation outcomes, total no of pulmonary embolism positive patients are 989, since 128 patients were diagnosed with pulmonary embolism after the intubation

^b^Total no of pulmonary embolism negative COVID-19 patients for primary outcomes (mortality and intubation) are 30,383. For secondary outcomes, total count for pulmonary embolism negative patients are 4468

^c^Primary outcomes are reported as adjusted HR (aHR), secondary outcomes are reported as Odds ratio (OR)

For the laboratory/monitoring parameters, PE COVID-19 patients had higher CRP (OR 1.001 [1.0009–1.002]) and LDH values (1.0003 [1.00001–1.0006])**;** however, no appreciable clinical difference was noted, as the values were mostly overlapping (Additional file 1: Fig. S3). The two groups did not significantly differ in ferritin (OR 1 [0.99–1]) or lymphocyte count (OR 1.04 [0.99–1.09]). The PE patients had decreased fibrinogen (OR 0.99 [0.998–0.99]) compared to the non-PE patients. Heart rate was slightly increased in PE patients (OR 1.009 [1.004–1.013]).

**Fig. 4:**
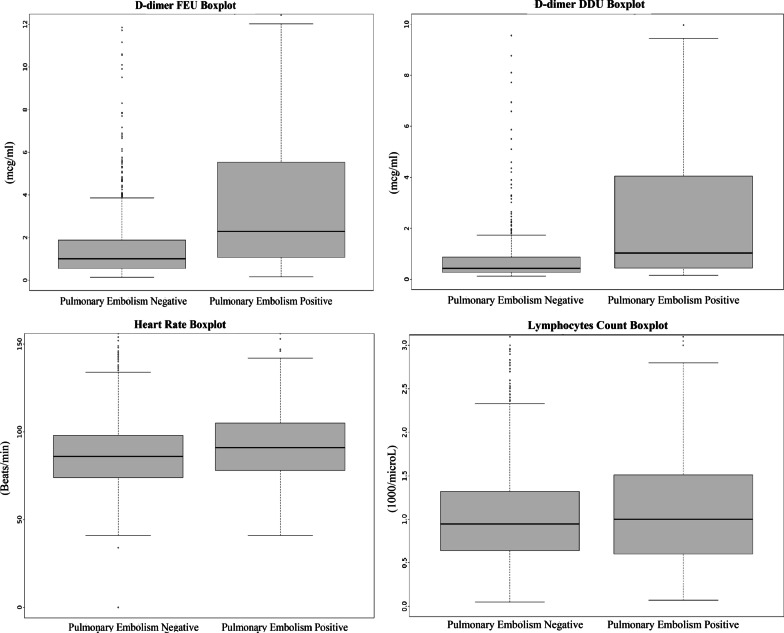
Box plot comparison of d-dimer FEU, d-Dimer DDU, Heart Rate and Lymphocytes Count in COVID-19 with and without Acute Pulmonary Embolism. Difference in d-dimer (FEU and DDU) in patients with and without pulmonary embolism can be clearly appreciated. Very slight difference is noted in heart rate. No difference is noted in the lymphocyte count. Out of 31,500 COVID-19 hospitalized patients, 1117(3.5%) of these patients were diagnosed with acute PE in this study

**Fig. 5:**
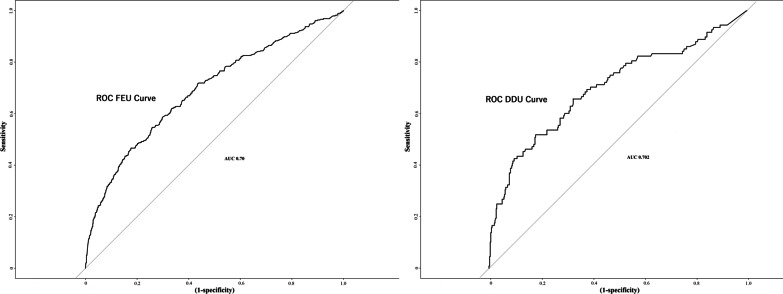
ROC curve for d-Dimer FEU and D d-Dimer DDU predicting acute pulmonary embolism in COVID-19 patients. Area under curve for both d-Dimer FEU and d-dimer DDU was 0.70

The admission d-dimer FEU and DDU measurements were recorded at median 0 [IQR0-1] days since admission. Statistically and clinically meaningful differences were noted in d-dimer in the PE patients, compared to non-PE patients (Fig. 4). PE patients had higher d-dimer FEU (OR 1.13 [1.1–1.15]) and d-dimer DDU (OR 1.13 [1.06–1.21]) values. In ROC curve analyses, d-dimer FEU and d-dimer DDU were noted to have reasonable AUCs (0.70 for both) (Fig. 5). A clinical calculator indicating the sensitivity, specificity, positive predictive value, and accuracy of d-dimer in predicting acute PE in COVID-19 was devised and published online by our group [21]. For d-Dimer FEU, at 1.8 mcg/ml, the test reached the sensitivity 55%, specificity 74%, positive predictive value 34%, and accuracy 70%. At 0.8 mcg/ml cut off, d-dimer DDU reached the sensitivity 54%, specificity 75%, positive predictive value 37%, and accuracy 70%. At higher cut-offs, 7 mcg/ml d-Dimer FEU and 3.5mcg/ml d-dimer DDU, specificity and accuracy improved to 95% and 81%, respectively, at the expense of poor sensitivity (≤ 25%) (Additional file 1: Tables S2, S3).

## Discussion

In the large population-based national cohort in the US, we report worse mortality and morbidity outcomes associated with the diagnosis of acute PE in hospitalized COVID-19 patients. D-dimer was noted to have utility in predicting pulmonary embolism starting at the cut-off levels 1.8 mcg/ml D-dimer FEU and 0.8 mcg/ml d-dimer DDU. We also present an admission d-dimer based calculator as a prediction tool for diagnosing acute PE in hospitalized COVID-19 patients.

The incidence of acute PE in hospitalized COVID-19 patients was noted at 3.5% in our study, which is lower than has been recorded in previous studies. In the French multicenter study of 1240 patients, the incidence of acute PE was noted at 8.7% in hospitalized COVID-19 patients; however, patients without CTPE (computerized axial tomography pulmonary embolism) imaging were excluded [6]. Similarly, the overall PE incidence of 9.5% in mechanically ventilated patients in our study is lower than has been previously reported for critically ill COVID-19 patients [25]. The lower incidence of PE in COVID-19 patients in our study represents the real-life clinical data, where*—*unlike protocol-driven studies*—*not all the patients are screened for acute PE. This points out the possibility of underdiagnosis of venous thromboembolism in this difficult-to-diagnose population. In our univariable analysis, COVID-19 patients with acute PE were more likely to have traditional risk factors (older age, obesity, history of malignancy) than patients without PE [26, 27]. Patients with PE were more likely to have cardiovascular risk factors; hypertension, diabetes, atrial fibrillation, and congestive heart failure [28, 29]. PE patients were sicker with a higher incidence of cerebrovascular diseases, acute encephalopathy, and sepsis. A high proportion of COVID-19 patients had acute kidney injury and chronic kidney injury (Table 1), more so in PE patients, which reflects upon the real-life dilemma that clinicians have to face when balancing the need for frequent image screening of PE against the high likelihood of contrast-induced kidney injury in this population.

Previous studies have noted increased mechanical ventilation rates consistent with the results of our study, but they have been underpowered to detect mortality differences [5, 6]. We report a clear association of mortality with PE diagnosis in COVID-19 patients. PE was mostly diagnosed earlier on during the admission and at the time of clinical deterioration during the peri-intubation period (Additional file 1: Fig. S2). Increased hospital length of stay was associated with the PE diagnosis. This underscores the importance of diagnosing acute PE in COVID-19 patients, which has an impact on important clinical outcomes.

Higher admission D-dimers of statistical and clinical significance were noted in PE patients than non-PE COVID-19 patients. Previous studies have reported an association of high D-dimer levels with increased mortality in COVID-19, which may be partly explained by the increased incidence of acute PE in patients with high d-dimer as noted in our study [30, 31].The close relationship of d-dimer levels with acute PE has been previously reported; however, it has not been established as a predictive risk tool for the evaluation of PE in COVID-19 [5, 32, 33]. Earlier smaller reports have reported variable d-dimer cut-off’s (ranging from 1 mcg/ml to 7.5 mcg/ml) to predict PE. However, the small size of the studies limits the ability to ascertain accurate prediction analysis of d-dimer [34–38]. In our study, at cut off values 1.8 mcg/ml d-dimer FEU and 0.8 mcg/ml d-dimer DDU, the test began to have clinical utility (accuracy 70%) for predicting acute PE in COVID-19 with specificity recorded at 74% for d-dimer FEU and 75% for d-dimer DDU. However, since the sensitivity was low (55% for d-dimer FEU and 54% for d-dimer DDU) at these cut-off values, the possibility of acute PE below the cut-off values cannot be ruled out. As the d-dimer values further increased, d-dimer had better ability to discriminate PE—specificity, positive predictive value, and accuracy improved—from the expected elevation in the setting of COVID-19 hospitalization; however, sensitivity continued to drop (Additional file 1: Tables S2, S3). We thus found d-dimer to be helpful in predicting pulmonary embolism in COVID-19 at the cut-off values 1.8 mcg/ml d-dimer FEU and 0.8 mcg/ml d-dimer DDU. We devised a clinical calculator (listing sensitivity, specificity, positive predictive values, and accuracy) using the threshold values of d-dimers to predict the probability of acute PE in hospitalized COVID-19 patients [21].

Patients with PE were more likely to have a history of PE or deep vein thrombosis with a high odds ratio of 5.11 [3.75–6.98], suggesting a lower threshold of screening for PE in this patient population. Chest pain was also more frequent in patients with PE. CRP and LDH were significantly increased in PE patients with COVID-19. This may still point towards the slightly higher inflammation noted in PE patients, supporting the hypothesis of ‘thromboinflammation’ for PE development [39, 40]. Slightly lower fibrinogen levels were noted in PE patients, as has been described before [41]. The heart rate differences pointed out between the two groups were not of clinical significance or usefulness (Fig. 4).

Our study has several limitations. As would be the case with any observational electronic health database study, the data retrieved via chart analysis may be subject to inaccuracies such as documentation errors. The number of patients that were screened with CT PE could not be retrieved due to the limitations of the database. The data may include patients who have not had the time to age into the outcome measurement yet, as the data is being regularly retrieved from the original sites. We adjusted for known confounders in our study, but the possibility of residual confounding exists, although sensitivity analysis indicated that addition of further covariates will not significantly affect the results (Additional file 1: Methods, Table S4).

There are several strengths of our study. We report by far the largest cohort analyzed for acute pulmonary embolism in COVID-19 patients from a geographically dispersed and demographically diverse multicenter population in the US. This allowed us sufficient power to delineate the effect of PE on COVID-19 mortality. The diagnosis of acute PE in COVID-19 is fraught with difficulty. For the first time, we provide a d-dimer based clinical calculator for predicting the probability of the diagnosis. The predictive risk tool underscores the utility and limitations of d-dimer at different threshold levels. We further defined optimal cut-offs where the utility of d-dimers is observed in predicting pulmonary embolism in hospitalized COVID-19 patients.

In conclusion, we report increased mortality and mechanical ventilation rates with acute PE in COVID-19 patients. d-dimer had potential utility in predicting pulmonary embolism in hospitalized COVID-19 patients. Further prospective studies should be performed to validate the pulmonary embolism risk model presented in our study.

## Supplementary Information

**Additional file 1**: **Fig S1**. Standardized Mean Differences of covariates before and after propensity score matching in the main analysis; Dot-plot Graph. The clinical conditions and the treatments are well-aligned. **Fig S2**. Temporal Relationship of Pulmonary Embolism diagnosis to Intubation. **Fig S3**. Box-plot comparison of CRP, Ferritin, Fibrinogen and LDH Count in COVID-19 with and without Acute Pulmonary Embolism. **Table S1**. Patient distribution in main and subgroup 1:4 propensity score-matched analysis. **Table S2**. Testing D-dimer FEU as a diagnostic test for Acute Pulmonary Embolism in COVID-19 for different cut off values. **Table S3**. Testing D dimer DDU as a diagnostic test for Acute Pulmonary Embolism in COVID-19 for different cut off value. **Table S4**: Sensitivity Analysis, outcomes of COVID-19 patients with and with-out acute pulmonary embolism after addition of three covariates; acute encephalopathy, stroke, and DVT. **Table S5**. Recurrent Mortality Analysis with Variable Censoring Times (Primary Analysis Data). **Table S6**. Recurrent Mortality Analysis with Variable Censoring Times (Sensitivity Analysis Data). **Table S7**. Recurrent Intubation analysis with Variable Censoring Times (Primary Analysis Data). **Table S8**. Recurrent Intubation Analysis with Variable Censoring Times (Sensitivity Analysis Data). **Table S9**. Diagnostic Codes for Clinical Conditions.

**Additional file 2**: Appendix B.

## Data Availability

The data repository is located at the N3C Data Enclave. An institutional database agreement needs to be in place prior to joining the N3C for level II access which is required for data-sharing. Will individual participant data be available (including data dictionaries)?, All data has been de-identified in the N3C database. What data in particular will be shared?, Access to the de-identified database can be shared upon joining the N3C. What other documents will be available?, Not applicable. When will data be available (start and end dates)?, Immediately following the publication to one year post-publication (the project is hosted on the N3C enclave platform for one year). With whom?, Investigator who has institutional data use agreement with N3C. For what types of analyses?, Any purpose. By what mechanism will data be made available?, Data is accessible on the N3C enclave platform with the institutional database agreement.

## Abbreviations

PE: Pulmonary embolism
CTPE: Computerized axial tomography pulmonary embolism
N3C: National Collaborative COVID-19 cohort
LDH: Lactate dehydrogenase
CRP: C-reactive protein
ROC: Receiver operative characteristic
AUC: Area under curve
FEU: Fibrinogen equivalent unit
DDU: D-dimer units
aHR: Adjusted hazard ratio
HR: Hazard ratio
OR: Odds ratio
IQR: Interquartile range
