# Supplementary material for: Predictors and outcomes of acute pulmonary embolism in COVID-19; insights from US National COVID cohort collaborative
Source: Respir Res. 2023 Feb 21;24:59. doi: 10.1186/s12931-023-02369-7 (PMC9942071; doi:10.1186/s12931-023-02369-7)
Supplement: Supplementary file 1 — Additional file 1: Fig S1. Standardized Mean Differences of covariates before and after propensity score matching in the main analysis; Dot-plot Graph. The clinical conditions and the treatments are well-aligned. Fig S2. Temporal Relationship of Pulmonary Embolism diagnosis to Intubation. Fig S3. Box-plot comparison of CRP, Ferritin, Fibrinogen and LDH Count in COVID-19 with and without Acute Pulmonary Embolism. Table S1. Patient distribution in main and subgroup 1:4 propensity score-matched analysis. Table S2. Testing D-dimer FEU as a diagnostic test for Acute Pulmonary Embolism in COVID-19 for different cut off values. Table S3. Testing D dimer DDU as a diagnostic test for Acute Pulmonary Embolism in COVID-19 for different cut off value. Table S4: Sensitivity Analysis, outcomes of COVID-19 patients with and with-out acute pulmonary embolism after addition of three covariates; acute encephalopathy, stroke, and DVT. Table S5. Recurrent Mortality Analysis with Variable Censoring Times (Primary Analysis Data). Table S6. Recurrent Mortality Analysis with Variable Censoring Times (Sensitivity Analysis Data). Table S7. Recurrent Intubation analysis with Variable Censoring Times (Primary Analysis Data). Table S8. Recurrent Intubation Analysis with Variable Censoring Times (Sensitivity Analysis Data). Table S9. Diagnostic Codes for Clinical Conditions. [file 12931_2023_2369_MOESM1_ESM.docx]

**Additional file 1: Appendix A**

**Additional file Source information**

**Additional file Statistical Methods**

**Fig S1. Standardized Mean Differences of covariates before and after propensity score matching in the main analysis; Dot-plot Graph**

The clinical conditions and the treatments are well-aligned.

**Fig S2. Temporal Relationship of Pulmonary Embolism diagnosis to Intubation**

**Fig S3. Box-plot comparison of CRP, Ferritin, Fibrinogen and LDH Count in COVID-19 with and without Acute Pulmonary Embolism**

**Table S1. Patient distribution in main and subgroup 1:4 propensity score-matched analysis**

**Table S2. Testing D-dimer FEU as a diagnostic test for Acute Pulmonary Embolism in COVID-19 for different cut off values**

**Table S3. Testing D dimer DDU as a diagnostic test for Acute Pulmonary Embolism in COVID-19 for different cut off value**

**Table S4: Sensitivity Analysis, outcomes of COVID-19 patients with and with-out acute pulmonary embolism after addition of three covariates; acute encephalopathy, stroke, and DVT**

**Table S5. Recurrent Mortality Analysis with Variable Censoring Times (Primary Analysis Data)**

**Table S6.** **Recurrent Mortality Analysis with Variable Censoring Times (Sensitivity Analysis Data)**

**Table S7. Recurrent Intubation analysis with Variable Censoring Times (Primary Analysis Data)**

**Table S8. Recurrent Intubation Analysis with Variable Censoring Times (Sensitivity Analysis Data)**

**Table S9. Diagnostic Codes for Clinical Conditions**

**Data Source information**

The N3C provides ethnically diverse, granular data from multiple clinical sites throughout the US. The details of the N3C cohort definition are publicly available on GitHub^1^. The dataset continues to grow as more patients from existing, and new clinical sites continue to be added to the database. Updated data payloads are sent by contributing sites approximately every 1-2 weeks. It is encouraged for the clinical sites to use the N3C COVID-19 phenotype to have a consistent cohort definition across the N3C dataset. N3C accepts data in the native common data model (CDM) format, be it OMOP, PCORnet, ACT, or TriNETX. N3C harmonizes its site data into Observational Medical Outcomes (OMOP) in conjunction with subject data experts from the CDM community. Through this method, clinical diagnosis, medication, observations, vital signs, and procedures are mapped into OMOP vocabulary. N3C ensures HIPAA compliance of the clinical patient data. Patient identifiers are synthetic and meant for research purposes. Furthermore, the dataset has been anonymized to ensure that patients cannot be individually identified. The site data, which has been checked through a robust quality assessment process, is added to the database for N3C community use.

References

E1. Github. Phenotype Data Acquisition. 6/27/2021. Available from: <https://github.com/National-COVID-Cohort-Collaborative/Phenotype_Data_Acquisition>.

**Additional file Statistical Methods**

The assumption in the Cox Model was the proportionality of hazards. This was tested individually for each covariate using Log minus Log survival plot (LML). If the curves were meeting each other on the LML plot, the assumption of proportionality of hazards was thought to be violated. For continuous variable (age), Schonfeld residual plot was constructed. If the 95% confidence intervals of the line were not within the zero residual in the plot, the proportionality of hazards assumption was not met. Age, race, nicotine dependence, malignancy, DVT, stroke, and all the treatments (steroids, remdesivir, tocilizumab, hydroxychloroquine) had evidence in line with the non-proportionality of hazard assumption for the mortality outcome on the plot analysis. Only age and malignancy had proof in favor of non-proportionality in the intubation hazard assumption analysis.

The assumption of non-proportionality was then further evaluated by calculating hazard ratios at multiple different censor points (2,5,10,15,30,45 and 60 days) during the observation period. The hazard ratios remained stable throughout the observation period at all the censor points in the mortality and intubation analysis, using the primary and sensitivity analysis data, suggesting reasonable compatibility with the proportionality of hazard assumption. ( Table E5-E8 in online Suppl)

Sensitivity analysis was performed by adding three additional covariates; DVT, stroke and acute encephalopathy in the primary and secondary analysis model. These three covariates were not included in the main analysis, as they had the potential to be collider variables or strong instruments in certain clinical situations. For instance, DVT could act as a strong instrument in PE mortality analysis as it influences mortality via PE. Similarly PE is a major contributor towards mortality after stroke, thus stroke can act as a strong instrument in PE mortality analysis.^2^ PE in COVID-19 can result in encephalopathy via hypoxia and post mechanical ventilation, encephalopathy is common, thus encephalopathy could act as a collider variable in the PE-mechanical ventilation analysis. In all these clinical situations, these variables may not serve as true confounders and may not need to be adjusted. As such these kind of variables can be difficult to ascertain in an analysis, we thus performed sensitivity analysis to confirm that addition of such variables in our analysis should not change the results significantly. We compared the sensitivity analysis results to the original analysis results, by observing for any change in the significance of the results (Table E4 in online Suppl).

D-dimer DDU is a separate method of D-dimer assay analysis used by certain health systems, although D-dimer FEU is more commonly used. We used D-dimer DDU as a validation cohort for the D-dimer FEU (training cohort). We compared the ROC curve analysis of the D-dimer DDU with D-dimer FEU (Suppl Fig E4 and Suppl Fig 5). The ROC curves for both D-dimer assays were alike and the AUC was 0.7 for both, which thus provided the internal validation for the D-dimer FEU analysis.

Caliper of 0.2 was used in our propensity score matched analysis as it was the optimal caliper width for our analysis, as is evident from Fig E1, where all the covariates are well-balanced. 0.2 caliper has also been previously suggested as optimal caliper width.^3 4^

References

1. Github. Phenotype Data Acquisition [Available from: <https://github.com/National-COVID-Cohort-Collaborative/Phenotype_Data_Acquisition> accessed 6/27/2021.

2. Keller K, Hobohm L, Münzel T, et al. Impact of pulmonary embolism on in-hospital mortality of patients with ischemic stroke. *Journal of the Neurological Sciences* 2020;419:117174. doi: <https://doi.org/10.1016/j.jns.2020.117174>

3. Austin PC. Optimal caliper widths for propensity-score matching when estimating differences in means and differences in proportions in observational studies. *Pharm Stat* 2011;10(2):150-61. doi: 10.1002/pst.433 [published Online First: 2010/10/07]

4. Wang Y, Cai H, Li C, et al. Optimal Caliper Width for Propensity Score Matching of Three Treatment Groups: A Monte Carlo Study. *PLOS ONE* 2013;8(12):e81045. doi: 10.1371/journal.pone.0081045

**Fig S1. Standardized Mean Differences of covariates before and after propensity score matching in the main analysis; Dot-plot Graph**


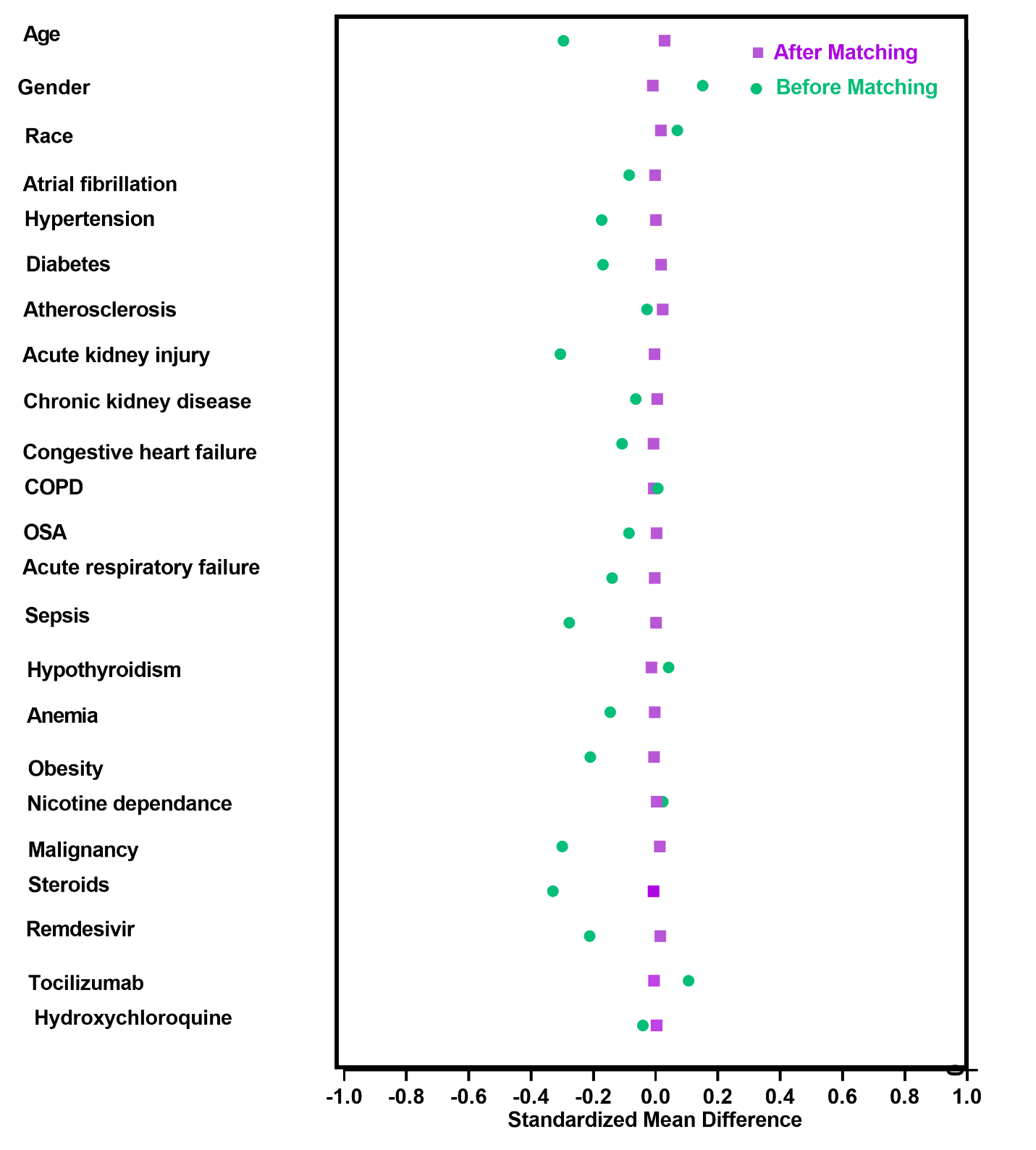


**Fig S2. Temporal Relationship of Pulmonary Embolism diagnosis to Intubation**

**
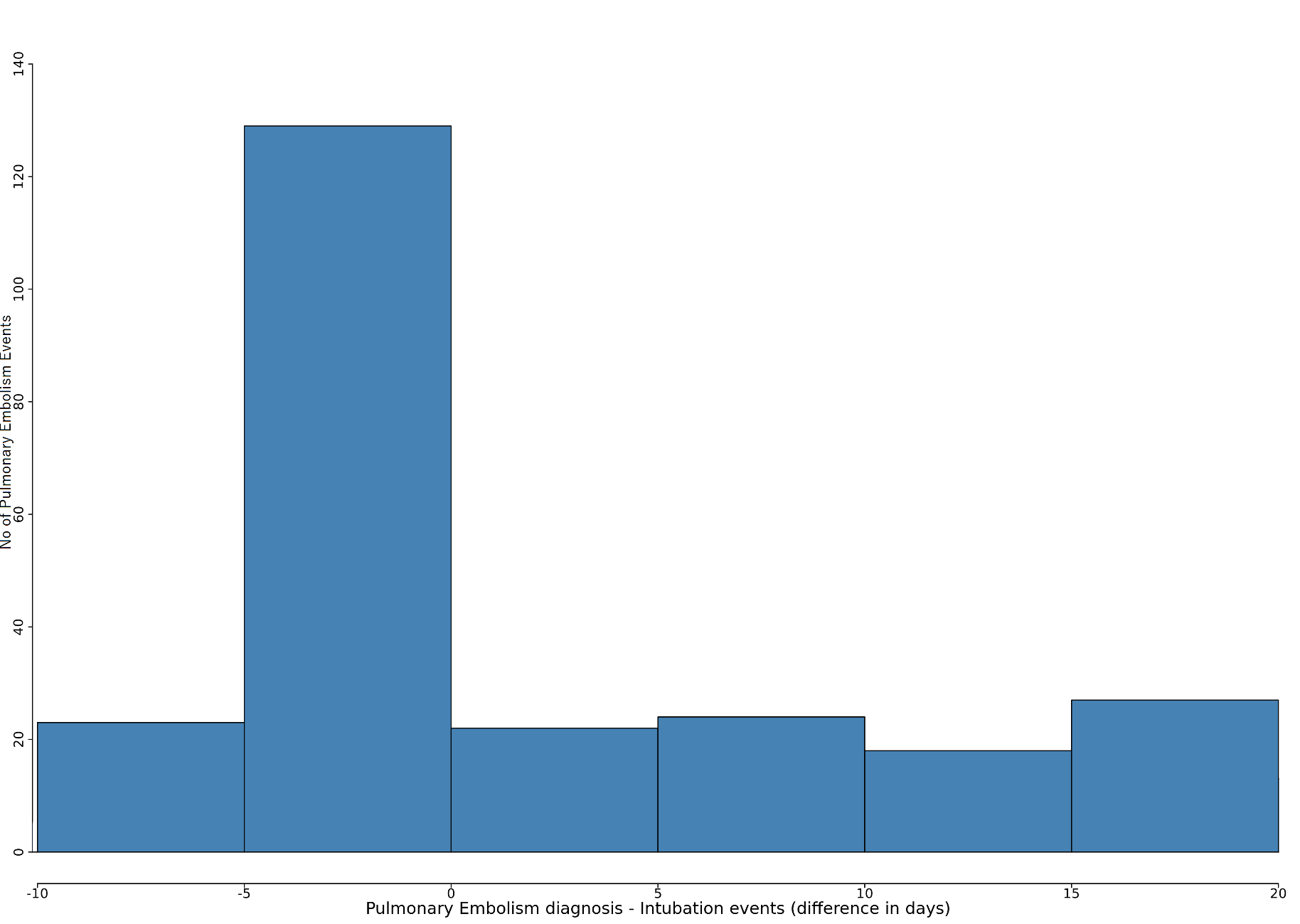
**

**Fig S3. Box plot comparison of CRP, Ferritin, Fibrinogen, and LDH Count in COVID-19 with and without Acute Pulmonary embolism**

**
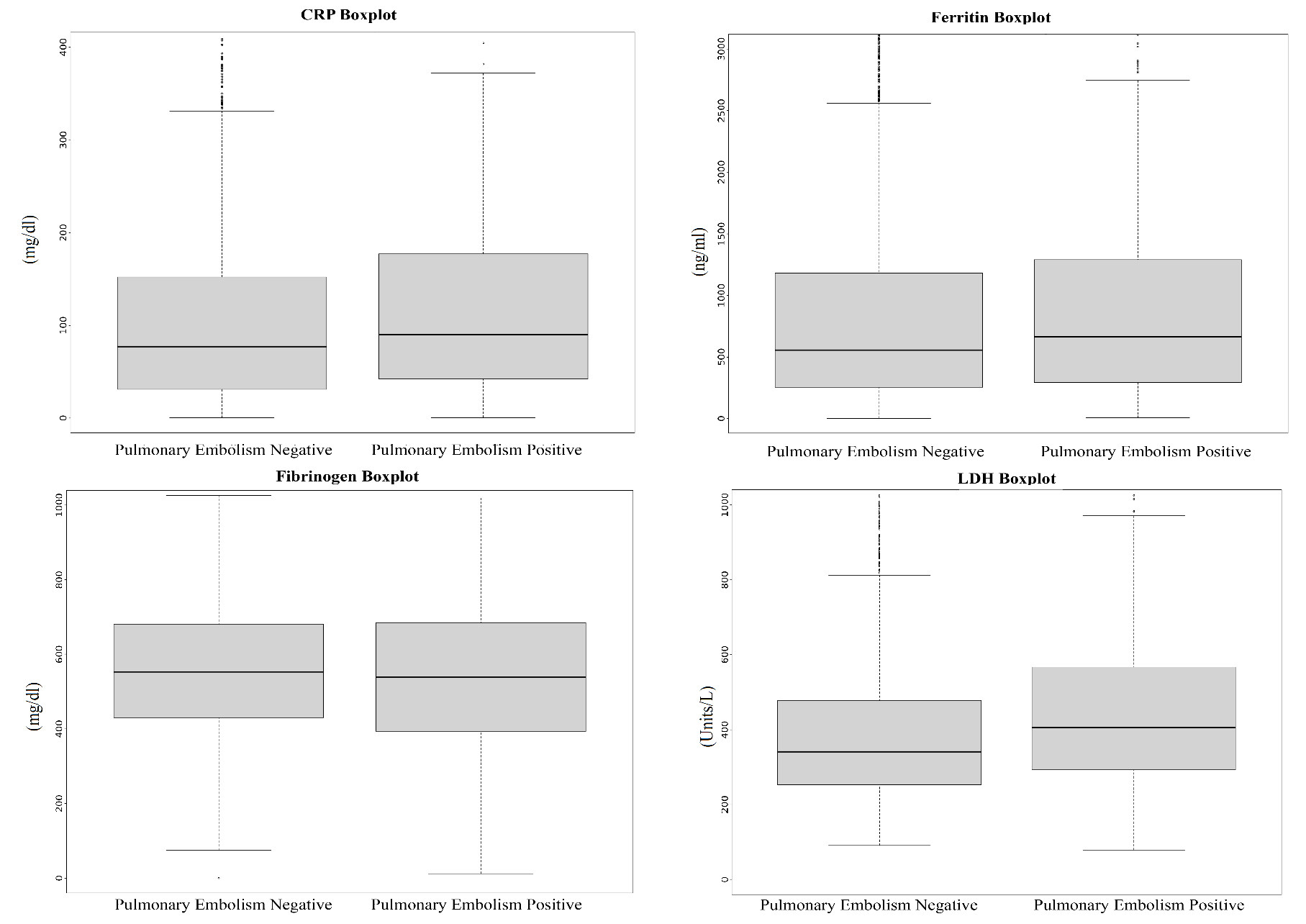
**

**Table S1. Patient distribution in main and subgroup 1:4 propensity score-matched analysis**

|  | **Pulmonary Embolism** | **Non-Pulmonary Embolism** |
| --- | --- | --- |
| **Main Analysis** | 1117 | 4468 |
| **CRP** | 612 | 2448 |
| **D-Dimer FEU** | 384 | 1536 |
| **D-dimer DDU** | 108 | 432 |
| **Ferritin** | 685 | 2740 |
| **Fibrinogen** | 369 | 1476 |
| **LDH** | 537 | 2148 |
| **Lymphocytes** | 353 | 1412 |
| **Heart Rate** | 576 | 2304 |

**Table S2. Testing D-dimer FEU as a diagnostic test for Acute Pulmonary Embolism in COVID-19 for different cut off values**

| **Value (mcg/ml)** | **Sensitivity (%)** | **Specificity(%)** | **Positive Predictive value (%)** | **Accuracy(%)** |
| --- | --- | --- | --- | --- |
| 0.5 | 90 | 21 | 22 | 34 |
| 0.6 | 86 | 28 | 23 | 40 |
| 0.7 | 83 | 36 | 24 | 45 |
| 0.8 | 81 | 41 | 25 | 48 |
| 0.9 | 76 | 46 | 26 | 53 |
| **1** | **74** | **50** | **27** | **55** |
| 1.1 | 72 | 55 | 28 | 58 |
| 1.2 | 69 | 57 | 29 | 60 |
| 1.3 | 65 | 61 | 30 | 62 |
| 1.4 | 62 | 66 | 31 | 65 |
| 1.5 | 60 | 67 | 31 | 65 |
| 1.6 | 59 | 70 | 32 | 67 |
| 1.7 | 58 | 71 | 33 | 68 |
| 1.8 | 55 | 74 | 34 | 70 |
| 1.9 | 51 | 75 | 34 | 71 |
| **2** | **49** | **76** | **35** | **71** |
| 2.1 | 49 | 78 | 36 | 72 |
| 2.2 | 49 | 79 | 37 | 73 |
| 2.3 | 48 | 80 | 37 | 74 |
| 2.4 | 47 | 81 | 38 | 74 |
| 2.5 | 46 | 82 | 39 | 75 |
| 2.6 | 45 | 83 | 40 | 75 |
| 2.7 | 44 | 83 | 40 | 76 |
| 2.8 | 43 | 84 | 40 | 76 |
| 2.9 | 43 | 84 | 41 | 76 |
| **3** | **43** | **84** | **41** | **76** |
| 3.1 | 42 | 85 | 41 | 76 |
| 3.2 | 42 | 86 | 42 | 77 |
| 3.3 | 41 | 86 | 42 | 77 |
| 3.4 | 40 | 86 | 43 | 77 |
| 3.5 | 39 | 87 | 43 | 77 |
| 3.6 | 38 | 87 | 43 | 77 |
| 3.7 | 37 | 88 | 43 | 78 |
| 3.8 | 36 | 88 | 44 | 78 |
| 3.9 | 36 | 89 | 45 | 78 |
| **4.0** | **34** | **89** | **45** | **78** |
| 4.1 | 34 | 90 | 45 | 78 |
| 4.2 | 33 | 90 | 45 | 79 |
| 4.3 | 33 | 90 | 45 | 79 |
| 4.5 | 32 | 79 | 47 | 79 |
| 4.6 | 32 | 91 | 47 | 79 |
| 4.7 | 32 | 91 | 48 | 79 |
| 4.8 | 31 | 92 | 48 | 79 |
| 4.9 | 30 | 92 | 48 | 80 |
| **5** | **29** | **92** | **49** | **80** |
| **Value (mcg/ml)** | **Sensitivity (%)** | **Specificity(%)** | **Positive Predictive Value (%)** | **Accuracy(%)** |
| 5.1 | 29 | 92 | 49 | 80 |
| 5.2 | 28 | 92 | 49 | 80 |
| 5.3 | 28 | 93 | 49 | 80 |
| 5.4 | 28 | 93 | 49 | 80 |
| 5.5 | 27 | 93 | 49 | 80 |
| 5.6 | 27 | 93 | 50 | 80 |
| 5.7 | 27 | 93 | 50 | 80 |
| 5.8 | 26 | 93 | 50 | 80 |
| 5.9 | 26 | 93 | 50 | 80 |
| **6.0** | **26** | **93** | **51** | **80** |
| 6.1 | 25 | 94 | 51 | 80 |
| 6.2 | 25 | 94 | 52 | 80 |
| 6.3 | 25 | 94 | 52 | 80 |
| 6.4 | 25 | 94 | 52 | 80 |
| 6.5 | 24 | 94 | 52 | 80 |
| 6.6 | 24 | 94 | 52 | 80 |
| 6.7 | 24 | 94 | 53 | 80 |
| 6.8 | 24 | 94 | 53 | 80 |
| 6.9 | 24 | 95 | 54 | 81 |
| **7** | **24** | **95** | **55** | **81** |
| 7.1 | 24 | 95 | 55 | 81 |
| 7.2 | 23 | 95 | 55 | 81 |
| 7.3 | 23 | 95 | 55 | 81 |
| 7.4 | 23 | 95 | 55 | 81 |
| 7.5 | 23 | 95 | 55 | 81 |
| 7.6 | 23 | 95 | 55 | 81 |
| 7.7 | 23 | 95 | 55 | 81 |
| 7.8 | 23 | 95 | 55 | 81 |
| 7.9 | 23 | 96 | 56 | 81 |
| **8** | **22** | **96** | **56** | **81** |
| 8.1 | 22 | 96 | 56 | 81 |
| 8.2 | 22 | 96 | 56 | 81 |
| 8.3 | 22 | 96 | 56 | 81 |
| 8.4 | 22 | 96 | 56 | 81 |
| 8.5 | 22 | 96 | 56 | 81 |
| 8.6 | 22 | 96 | 57 | 81 |
| 8.7 | 22 | 96 | 57 | 81 |
| 8.8 | 22 | 96 | 58 | 81 |
| 8.9 | 21 | 96 | 58 | 81 |
| **9** | **21** | **96** | **58** | **81** |
| 9.1 | 21 | 96 | 58 | 81 |
| 9.2 | 21 | 96 | 58 | 81 |
| 9.3 | 20 | 96 | 58 | 81 |
| 9.4 | 20 | 96 | 58 | 81 |
| 9.5 | 20 | 96 | 58 | 81 |
| 9.6 | 20 | 96 | 58 | 81 |
| **Value (mcg/ml)** | **Sensitivity (%)** | **Specificity(%)** | **Positive Predictive Value (%)** | **Accuracy(%)** |
| 9.8 | 20 | 96 | 58 | 81 |
| 9.9 | 20 | 96 | 58 | 81 |
| **10** | **20** | **96** | **59** | **81** |

**Table S3. Testing D dimer DDU as a diagnostic test for Acute Pulmonary Embolism in COVID-19 for different cut off values**

| **Value (mcg/ml)** | **Sensitivity (%)** | **Specificity(%)** | **Positive Predictive Value (%)** | **Accuracy(%)** |
| --- | --- | --- | --- | --- |
| 0.3 | 83 | 32 | 23 | 42 |
| 0.4 | 79 | 47 | 27 | 53 |
| **0.5** | **71** | **57** | **29** | **60** |
| 0.6 | 66 | 66 | 33 | 66 |
| 0.7 | 58 | 72 | 34 | 69 |
| 0.8 | 54 | 75 | 37 | 70 |
| 0.9 | 51 | 77 | 39 | 72 |
| **1** | **52** | **81** | **40** | **75** |
| 1.1 | 46 | 83 | 40 | 75 |
| 1.2 | 46 | 85 | 43 | 77 |
| 1.3 | 43 | 86 | 45 | 78 |
| 1.4 | 43 | 87 | 47 | 79 |
| **1.5** | **43** | **87** | **47** | **78** |
| 1.6 | 43 | 88 | 48 | 79 |
| 1.7 | 43 | 88 | 49 | 79 |
| 1.8 | 42 | 89 | 49 | 80 |
| 1.9 | 42 | 89 | 50 | 80 |
| **2** | **42** | **90** | **52** | **80** |
| 2.1 | 41 | 90 | 52 | 80 |
| 2.2 | 39 | 91 | 52 | 80 |
| 2.3 | 38 | 92 | 53 | 80 |
| 2.4 | 35 | 92 | 53 | 80 |
| **2.5** | **32** | **92** | **51** | **80** |
| 2.6 | 32 | 93 | 53 | 81 |
| 2.7 | 31 | 93 | 54 | 81 |
| 2.8 | 31 | 93 | 55 | 81 |
| 2.9 | 31 | 93 | 55 | 81 |
| **3** | **29** | **93** | **54** | **81** |
| 3.1 | 28 | 94 | 54 | 81 |
| 3.2 | 28 | 94 | 54 | 81 |
| 3.3 | 26 | 94 | 55 | 81 |
| 3.4 | 26 | 95 | 56 | 81 |
| **3.5** | **25** | **95** | **55** | **81** |
| 3.6 | 25 | 95 | 56 | 81 |
|  |  |  |  |  |
| 3.8 | 25 | 96 | 60 | 82 |
| 3.9 | 25 | 96 | 60 | 81 |
| **4** | **25** | **96** | **60** | **82** |
| 4.1 | 25 | 96 | 61 | 82 |
| 4.2 | 25 | 96 | 61 | 82 |
| 4.3 | 25 | 96 | 63 | 82 |
| 4.4 | 25 | 96 | 64 | 82 |
| **4.5** | **25** | **97** | **66** | **82** |
| **Value (mcg/ml)** | **Sensitivity (%)** | **Specificity(%)** | **Positive Predictive Value (%)** | **Accuracy(%)** |
| 4.7 | 23 | 97 | 66 | 82 |
| 4.8 | 23 | 97 | 67 | 82 |
| 4.9 | 23 | 97 | 67 | 82 |
| **5** | **22** | **97** | **66** | **82** |

**Table S4: Sensitivity Analysis, outcomes of COVID-19 patients with and with-out acute pulmonary embolism after addition of three covariates; acute encephalopathy, stroke, and DVT**

|  | **Pulmonary Embolism Positive*** | **Pulmonary Embolism Negative**** | **aHR/OR,**  **p values^¥^** |
| --- | --- | --- | --- |
| **Mortality** | 264(23.6%) | 3918(12.8%) | **1.34(1.18-1.53),**  **p<0.001** |
| **Intubation** | 174 (17.6%) | 2874(9.3%) | **1.29 (1.11-1.52),**  **p=0.001** |
| **History of Pulmonary Embolism/DVT** | 92(8.2%) | 89(2%) | **4.41(3.27-5.95),**  **p<0.001** |
| **Chest pain** | 93(8.3%) | 262(5.9%) | **1.45 (1.13-1.86),**  **p=0.002** |
| **Length of stay**  **(days)** | 9(5-18) | 6(3-12) | **1.025(1.02-1.03),**  **p<0.001** |
| **CRP**  **(mg/dl)** | 92(42-176.2) | 80.5(36-154) | **1.001(1.0004-1.002),**  **p=0.004** |
| **Ferritin**  **(ng/ml)** | 656(290-1292) | 608(286-1245) | **1.00001(0.99-1.00005),**  **p=0.42** |
| **D-dimer FEU**  **(mcg/ml)** | 1.98(0.97-6.4) | 1.045(0.57-2.06) | **1.09(1.07-1.11),**  **p<0.001** |
| **D-dimer DDU**  **(mcg/ml)** | 1.03(0.41-3.04) | 0.45(0.25-0.94) | **1.09 (1.04-1.14),**  **p<0.001** |
| **Fibrinogen**  **(mg/dl)** | 539(393-686.5) | 568(449.5-692) | **0.998(0.998-0.999),**  **p<0.001** |
| **LDH**  **(Units/L)** | 406(294-568) | 344(264-481) | **1.0009(1.0006-1.0012),**  **p<0.001** |
| **Lymphocytes**  **(1000/ml)** | 1(0.63-1.44) | 0.9(0.61-1.4) | **1.014(0.98-1.04),**  **p=0.369** |
| **Heart Rate**  **(beats/min)** | 89(77-104) | 87(75-100) | **1.006(1.001-1.011),**  **p=0.006** |

Data are n (%) or median (IQR 25-75^th^ percentile). No change in the significance of the results was observed in the sensitivity analysis when compared to the original analysis.

*Total no of pulmonary embolism positive COVID-19 patients for mortality and secondary outcomes (history of pulmonary embolism/DVT, chest pain, length of stay, CRP, ferritin, D-dimer FEU and DDU, fibrinogen, LDH, lymphocytes, Heart rate) are 1117. For intubation outcomes, total no of pulmonary embolism positive patients are 989, since 128 patients were diagnosed with pulmonary embolism after the intubation.

**Total no of pulmonary embolism negative COVID-19 patients for primary outcomes (mortality and intubation) are 30,383. For secondary outcomes, total count for pulmonary embolism negative patients are 4468.

**^¥^** Primary outcomes are reported as adjusted HR (aHR), secondary outcomes are reported as Odds ratio (OR)

**Table S5. Recurrent Mortality Analysis with Variable Censoring Times (Primary Analysis Data)**

| **Censoring Time** | **Hazard Ratio** | **95% Confidence Interval** | **P value** |
| --- | --- | --- | --- |
| 2 days | 1.35 | 1.19-1.53 | <0.001 |
| 5 days | 1.35 | 1.19-1.53 | <0.001 |
| 10 days | 1.35 | 1.19-1.53 | <0.001 |
| 20 days | 1.35 | 1.18-1.52 | <0.001 |
| 30 days | 1.35 | 1.18-1.52 | <0.001 |
| 45 days | 1.35 | 1.18-1.52 | <0.001 |
| 60 days | 1.35 | 1.19-1.53 | <0.001 |

**Table S6.** **Recurrent Mortality Analysis with Variable Censoring Times (Sensitivity Analysis Data)**

| **Censoring Time** | **Hazard Ratio** | **95% Confidence Interval** | **P value** |
| --- | --- | --- | --- |
| 2 days | 1.33 | 1.17-1.52 | <0.001 |
| 5 days | 1.33 | 1.17-1.52 | <0.001 |
| 10 days | 1.33 | 1.17-1.51 | <0.001 |
| 20 days | 1.32 | 1.17-1.50 | <0.001 |
| 30 days | 1.32 | 1.17-1.50 | <0.001 |
| 45 days | 1.33 | 1.17-1.51 | <0.001 |
| 60 days | 1.33 | 1.17-1.52 | <0.001 |

**Table S7. Recurrent Intubation analysis with Variable Censoring Times (Primary Analysis Data)**

| **Censoring Time** | **Hazard Ratio** | **95% Confidence Interval** | **P value** |
| --- | --- | --- | --- |
| 2 days | 1.38 | 1.19-1.62 | <0.001 |
| 5 days | 1.38 | 1.18-1.61 | <0.001 |
| 10 days | 1.38 | 1.18-1.62 | <0.001 |
| 20 days | 1.38 | 1.18-1.61 | <0.001 |
| 30 days | 1.38 | 1.18-1.61 | <0.001 |
| 45 days | 1.38 | 1.18-1.62 | <0.001 |
| 60 days | 1.38 | 1.18-1.62 | <0.001 |

**Table S8. Recurrent Intubation Analysis with Variable Censoring Times (Sensitivity Analysis Data)**

| **Censoring Time** | **Hazard Ratio** | **95% Confidence Interval** | **P value** |
| --- | --- | --- | --- |
| 2 days | 1.29 | 1.11-1.52 | <0.001 |
| 5 days | 1.29 | 1.11-1.51 | <0.001 |
| 10 days | 1.29 | 1.11-1.52 | <0.001 |
| 20 days | 1.29 | 1.11-1.52 | <0.001 |
| 30 days | 1.29 | 1.11-1.52 | <0.001 |
| 45 days | 1.29 | 1.11-1.52 | <0.001 |
| 60 days | 1.29 | 1.11-1.52 | <0.001 |

**Table S9. ICD-10-CM Diagnostic Codes for Clinical Conditions**

| **Acute Pulmonary Embolism** | “I26.02”, “I26.09”, “I26.92”, I26.93”, I26.94”, I26.99” |
| --- | --- |
| **Atrial Fibrillation/Flutter** | “I48” |
| **Essential hypertension** | “I10” |
| **Diabetes Mellitus** | “E08-E13” |
| **Atherosclerosis** | “I70” |
| **Acute Kidney Injury** | “N17” |
| **Chronic Kidney Disease** | “N18” |
| **Heart Failure** | “I-50” |
| **Chronic Obstructive Lung Disease** | “J44” |
| **Obstructive Sleep Apnea** | “G47.33” |
| **Sepsis** | “A41” |
| **Hypothyroidism** | “ E03”, “E89” |
| **Obesity** | “E66” |
| **Malignancy** | “C00-C96” |
| **Anemia** | “D50-D64” |
| **Nicotine Dependence** | “F17” |
| **Acute DVT of Extremities** | “I82.4”, “I82.6” |
| **History of Pulmonary Embolism/DVT** | “Z86.711”, **“**Z86.718” |
| **Stroke/Cerebrovascular diseases** | “I-60-I-69” |
| **Acute encephalopathy** | “G93.40-G93.41”, “G93.49” |
